# Influence of neuropathological diagnosis on psychooncological distress in neurooncological patients - a retrospective cross-sectional analysis

**DOI:** 10.3389/fonc.2024.1457017

**Published:** 2024-11-22

**Authors:** Franziska Staub-Bartelt, Sarah Obermayr, Michael Sabel, Marion Rapp

**Affiliations:** ^1^ Department of Neurosurgery, University Hospital Duesseldorf, Duesseldorf, Germany; ^2^ Department for Orthopaedics and Traumatology, Kufstein Bezirkskrankenhaus, Kufstein, Austria

**Keywords:** quality of life, glioma, neurooncology, mental health, distress

## Abstract

**Background:**

Gliomas, the most common primary brain tumours, are classified based on histology and molecular genetics. Glioblastomas (GBM) are highly aggressive and are graded as WHO grade 4, while astrocytoma and oligodendrogliomas fall under WHO grades 2-3 (4). Gliomas affect 6 per 100,000 people, with a higher incidence in men. GBM has the poorest prognosis, whereas grade 2 astrocytoma and oligodendrogliomas show better outcomes. Quality of life (QoL) is now a crucial therapeutic goal alongside survival. Despite the impact of gliomas on QoL, especially given their incurability and progressive neurological deficits, research specifically comparing QoL and psycho-oncological stress in GBM versus grade 2 gliomas (glioma_2) remains limited. This study aims to fill that gap using validated measurement methods.

**Methods:**

This retrospective, single-centre study investigated differences in QoL among neuro-oncological patients using the Karnofsky Performance Score (KPS), Distress Thermometer (DT), Hospital Anxiety and Depression Scale (HADS), and EORTC-QLQ-C30-BN20. Data were collected before chemotherapy or radiotherapy to avoid therapy impact on QoL. Out of 2258 patients screened until June 30, 2022, 639 had glioblastoma or WHO grade 2 gliomas, with 223 meeting inclusion criteria for analysis.

**Results:**

The study included 161 GBM and 62 Glioma_2 patients, with 64% of all patients being male. The mean age was 58.11 years (SD ± 16.186). The DT did not show significant differences between GBM and glioma_2 glioma patients (median GBM:6 vs. 5 in glioma_2, p=0.480). However, the HADS-D indicates that GBM patients experience significantly more depression (median GBM 4.5 vs. 4 in glioma_2, p=0.033), though anxiety levels are similar in both groups (median GBM. 6 vs. 6 in glioma_2, p=0.867). The KPS (median GBM 70 vs. 90 in glioma_2, p<0.001) and specific aspects of the EORTC-QLQ-C30-BN20 questionnaire demonstrate that GBM patients have notably greater physical impairments than glioma_2 patients at diagnosis. Overall, GBM patients report worse quality of life compared to glioma_2 patients (median GBM 50 vs. 67 in glioma_2, p<0.001).

**Conclusion:**

This study showed that distress is present in glioma patients regardless of their histopathological grading, even though GBM patients show higher depression levels and more physical limitations. Targeted anxiety management and early depression screening are essential for all glioma patients. Early QoL screening and making QoL a therapeutic goal benefits patient care and society.

## Introduction

Gliomas are the most common primary brain tumours, classified based on histology and molecular genetics. The actual WHO classification of CNS tumours, updated in 2021, emphasises molecular genetic factors and their implications for tumour aggressiveness and patient survival. Glioblastomas (GBM) are characterised by rapid, aggressive, and infiltrative growth and are assigned to WHO grade 4. Molecularly, GBM is distinguished from astrocytoma WHO grade 4 by the absence of an IDH mutation. Other common glioma groups include astrocytoma and oligodendroglioma, which are assigned WHO-grade 2-3 based on histology and specific factors. Both groups typically feature an IDH mutation, with oligodendrogliomas exhibiting a 1p/19q codeletion (1). Histopathological and molecular findings are of high therapeutical consequence for the patients. Survival times vary significantly depending on tumour type and WHO grade. Globally, the incidence of glioma is approximately 6 per 100,000 individuals, with men being 1.6 times more likely to be affected than women (2). The average age for GBM patients is around 65 years, depending on the study, while for low-grade glioma (LGG) patients, the average age is significantly lower, around 45 years, varying by subtype (3, 4). GBM, the most common malignant primary brain tumour (50%), has the poorest prognosis. A statistical report from the USA for 2016-2020 indicates a median survival of 8 months for GBM patients in a cohort of over 1000.000 people during 16 years, irrespective of whether individuals received any treatment for their tumour or not (3). In contrast, patients with WHO grade 2 astrocytoma, referred to as LGG (formerly diffuse astrocytoma), have a median survival of approximately 60 months, while oligodendroglioma patients have a median survival of about 199 months. The 5-year survival rate for glioblastoma patients is 7.2%, whereas 53.5% of patients with WHO grade 2 tumours show a 5-year survival. Only 4.7% of glioblastoma patients survive for 10 years, compared to 43.1% of WHO grade 2 astrocytoma patients and 69.6% of oligodendroglioma patients (3). Numerous studies have examined the survival rates of different tumour entities, with survival traditionally being the primary factor in oncological treatment planning.

However, quality of life (QoL) is increasingly recognised as an important therapeutic goal alongside survival and has become a focus of various studies (5–9). QoL encompasses both subjective and objective aspects such as health, autonomy, and freedom and is influenced by individual and environmental factors, including character, experiences, values, personal resources such as family support, social status and region of living (10–13). The personal prerequisites for good QoL can change dynamically over a person’s life. QoL can be negatively impacted by anxiety, burden, stress, distress and depression. Earlier publications proved that oncological patients commonly suffer from these negative influences, significantly reducing their QoL (14–16). Neurooncological, as a special subgroup of cancer patients, suffer from an incurable disease with increasing neurological deficits over time. Therefore, the impact on QoL is huge. Still, the burden differs between GBM and LGG patients, depending on the different therapy approaches, the different expected overall survival as well and the different life situations (regarding age, working situation, and family situation). However, literature that addresses this important and specific difference is sparse.

Few studies have analysed the impact of neuropathological tumour diagnosis on QoL and psycho-oncological stress (17–20). No study has yet used comprehensive measurement tools to compare the differences in QoL at the primary diagnosis of GBM versus LGG (WHO grade 2 gliomas). This work aims to gather previously unknown data on the burden and QoL of GBM patients and patients with WHO grade 2 gliomas using a representative study cohort and validated measurement methods and to analyse the differences in their psycho-oncological stress and QoL.

## Patients and methods

This study is a retrospective, single-centre investigation conducted at the Center for Neuro-Oncology in the Department of Neurosurgery at the University Hospital of Düsseldorf. Since 2010, patients have undergone screening for psycho-oncological distress and QoL using specific questionnaires. The study was approved by the Ethics Committee of Heinrich Heine University Düsseldorf under the file number 2022-1852.

To minimise potential bias effects from adjuvant radiation and chemotherapy on QoL, data were collected before treatment. For preoperative data, patients were aware of their suspected diagnosis, which was later confirmed by neuropathological findings.

Selection criteria for patients included treatment at the Centre for Neuro-Oncology at the University Hospital of Düsseldorf, a neuropathological confirmed diagnosis of WHO tumour grade 4 (GBM) or glioma WHO tumour grade 2 (Glioma_2), and no adjuvant therapy at the time of the survey. Exclusion criteria included receiving adjuvant therapy, multiple malignancies, and incomplete questionnaires.

The specific inclusion and exclusion criteria are outlined in the table below (**Table 1**):

**Table 1:** Patient selection and inclusion/exclusion criteria.

*Inclusion Criteria*	*Exclusion Criteria:*
Patient treated + in the Department of Neurosurgery at the University Hospital of Düsseldorf.	Patients who had already received adjuvant therapy/recurrent disease
Age ≥18 years.	Patients with multiple malignancies.
Provided informed consent.	Patients with more than half of the questionnaires incomplete.
Neuropathologically confirmed primary diagnosis of glioblastoma WHO tumour grade 4 or glioma WHO tumour grade 2 according to the current diagnostic criteria at the time of therapy.	Lack of cognitive understanding of the questions.
For glioma WHO tumour grade 2 patients: perioperative or follow-up data collection with stable disease (no clinical or radiological indication of recurrence).	Poor health status preventing them from answering the questions.
No adjuvant therapy received at timepoint of inclusion Cognitive ability to independently complete the questionnaires.	Lack of proficiency in German.

Overall, EORTC-QLQ-C30-BN20 questionnaires were available from 639 Patients diagnosed with either GBM or Glioma_2, who underwent the interview at any timepoint of diagnosis. 223 of these 639 (34.9%) patients had filled in the EORTC-QLQ-C30-BN20 questionnaire at the point of initial diagnosis and, therefore, met the predefined inclusion criteria.

Socioeconomic data such as gender, age, Karnofsky Performance Status (KPS), relationship status, psychiatric history, and others were obtained from hospital software “Medico” (CompuGroupMedical, CGM Clinical Europe GmbH).

### Data collection and questionnaires - MedForm App

For data collection on the psycho-oncological burden and QoL of patients, the “MedForm App” was used. This application was developed by Mr. Frank Escher in 2020 and is utilised on Samsung Galaxy Tab A (2016) tablets. MedForm is a user-friendly application that guides patients through various input pages, requesting basic personal information such as name, date of birth, nationality, and gender, as well as socioeconomic data like education level, marital status, occupation, number of children, and psychosocial support. Further questions involve disease-specific details (date of initial diagnosis, current diagnosis, disease status, and adjuvant therapy information). At last, patients proceed to answer questions from standardised questionnaires embedded in the app, which assess QoL and psycho-oncological burden. The questionnaires integrated into MedForm are validated tools for assessing QoL and psycho-oncological burden in cancer patients.

These include:

- EORTC QLQ-C30 (European Organization for Research and Treatment of Cancer Quality of Life Questionnaire-Core 30): A standardised questionnaire for evaluating the QoL in cancer patients.

The EORTC initially was released in 1986/87 as QLQ-C36, the current version (EORTC QLQ-C30 Version 3.0) includes 30 evaluable questions covering 15 aspects of quality of life. Each aspect is scored on a scale from 0 to 100% (21). In addition to the general cancer questionnaire, there are disease-specific modules. The QLQ-BN20 was designed for brain tumour patients, featuring 20 specific questions (22, 23).

- HADS (Hospital Anxiety and Depression Scale): An instrument for assessing anxiety and depression in hospital patients.

The S3 Guideline recommends HADS for screening psychological distress alongside the Distress Thermometer (DT) (24). It consists of 14 questions without somatic symptoms. The results provide separate scores for anxiety and depression, which can also be combined to give a general distress score, though this combined score is not used in this study due to the use of DT for general distress. Scores are interpreted in three ranges: 0-7 (normal), 8-10 (borderline), and 11+ (abnormal). A cut-off score of 8 increases sensitivity but reduces specificity, capturing more at-risk patients. While HADS cannot diagnose anxiety and depression solely based on self-reported symptoms, elevated scores suggest the need for further evaluation by a specialist.

- Distress Thermometer (DT).

The DT is a multidisciplinary self-assessment screening tool developed by the National Comprehensive Cancer Network (NCCN) in the USA (25). Patients indicate their distress level over the past week, including the current day, on an analogue scale depicted as a thermometer, ranging from 0 (no distress) to 10 (extreme distress). Scores ≥ 5 are considered elevated. Its validity has been confirmed through multiple correlations with the HADS (26). This study uses the recommended cut-off value of ≥5 for neuro-oncological patients.

These questionnaires and the DT cover a broad range of dimensions, including emotional well-being, social functioning, and general physical complaints.

- KPS

For further evaluation of the physical functioning and reflection of dependence on external help of patients, the Karnofsky Performance Status (KPS) was used, which assesses the physical functioning of patients, particularly their ability to work and care for themselves. The use of KPS allows for a standardised assessment of the overall health status of patients.

For further detailed information and sample illustrations of the questionnaires used, we refer to the **Supplementary Material**.

### Sample size and statistical analysis

The required sample size was calculated by statisticians at Heinrich-Heine University before retrospective data collection and analysis. Although a larger sample would enhance the study’s power, the cohort size is acceptable given the rarity of gliomas and the specific inclusion criteria and is representative compared to other studies.

Statistical analysis aimed to compare differences in QoL aspects between patients with GBM and those with Glioma_2. Most analyses used descriptive statistics, given the comparison between two groups on various QoL aspects. Dependent variables included the DT, HADS, and EORTC-QLQ-C30-BN20. The KPS was analysed as both a dependent and independent variable.

Initially, the distribution of variables was examined, confirming normal distribution only for age. KPS, DT, HADS, and EORTC-QLQ-C30-BN20 results did not exhibit normal distribution. Despite the ordinal nature of these outcomes, median, mean and standard deviation (SD) are reported for comparability with other studies.

For non-normally distributed independent variables, differences between diagnostic groups were assessed using the Mann-Whitney U test as a non-parametric alternative to the T-test. Significance was set at p=0.05. The effect size was calculated using Pearson’s correlation coefficient (r), with thresholds of 0.1-<0.3 for weak, 0.3-<0.5 for moderate, and ≥0.5 for strong effects according to Cohen’s criteria. Given only two comparison groups, Bonferroni correction was not deemed necessary. Percentages are reported using valid percentages from SPSS, excluding missing data. To examine confounders, the cohort was dichotomised by gender, psychiatric history or medication, children, and relationship status, and results were compared within each diagnostic group. For example, only male or female GBM patients were analysed for differences in DT scores. Some analyses were impractical due to small subgroup sizes. Additionally, the correlation between physical condition and measurement outcomes was analysed using Pearson correlation, linking instrument results with KPS scores. All statistical analyses were conducted using IBM SPSS Statistics 28.0.1.1.

## Results

The study included 161 GBM and 62 Glioma_2 patients, with 64% of all patients being male. The mean age was 58.11 years (standard deviation ± 16.186). GBM patients were, on average, 24.25 years older than Glioma_2 patients (p < 0.005). Administration of the EORTC-QLQ-C30-BN20 questionnaires occurred either perioperatively at initial diagnosis or during follow-up for Glioma_2 patients. Most questionnaires were completed preoperatively, with 49% of GBM patients and 10% of Glioma_2 patients participating at this stage. Follow-up assessments were more common among Gliom_2 patients. Out of the 223 surveyed patients, 34 (15%) reported having a pre-existing psychiatric condition or the use of psychotropic medication. This subset included 18 GBM patients and 16 Glioma_2 patients. Furthermore, 114 patients (51%) reported being in a partnership, while 35 patients (16%) were single or widowed. Regarding family structure, 97 patients (43%) indicated they had children, whereas 19 patients (8%) reported being childless. **Table 2** summarises details of the entire cohort, displaying the two subgroups, GBM and Glioma_2.

**Table 2:** Provides a detailed overview of the entire cohort, distinguishing between GBM and Glioma_2 diagnosis groups.

	Total cohort n = 223	GBM n = 161	Glioma_2 n = 62
Age (mean ± SD)	58.11 ± 16.2	64.86 ± 12	40.6 ± 12
Female	79/35.40%	53/32.9%	26/41.9%
Male	144/64.6%	108/67.1%	36/58.1%
GBM	161/72.2%		
Glioma_2	62/27.8%		
Timepoint of assessment			
Pre-OP	132/59.2%	110/68.3%	22/25.5%
Post-OP	63/28.3%	51/31.7%	12/19.4%
Follow-Up	28/12.6%	0/0%	28/45.2%
Psychological precondition			
yes	97/43.5%	71/44.1%	26/41.9%
Tumour localisation			
Right	105/47.1%	76/47.2%	29/46.8%
Left	100/44.8%	68/42.2%	32/51.6%
Multiple	18/8.1%	17/10.6%	1/1.6%
Relationship status			
In a relationship	114/51.1%	85/52.8%	29/46.8%
Single	35/15.7%	22/13.7%	13/21%
Children			
Yes	97/43.5%	71/44.1%	26/41.9%
No	19/8.5%	10/6.2%	9/14.5%
KPS known	220/98.7%	158/98.15%	62/100%
DT completed	213/95.5%	152/94.4%	61/98.4%
HADS-A completed	163/73.1%	114/70.8%	49/79.0%
HADS-D completed	163/73.1%	114/70.8%	49/79.0%
EORTC completed	223/100%	161/100%	62/100%

It includes measurements of various factors and confounders: age, gender, survey timing, tumour location, relationship status, and parenthood. The table presents data on absolute numbers, means, percentages, and standard deviations (SD).

### KPS

**Figure 1:**
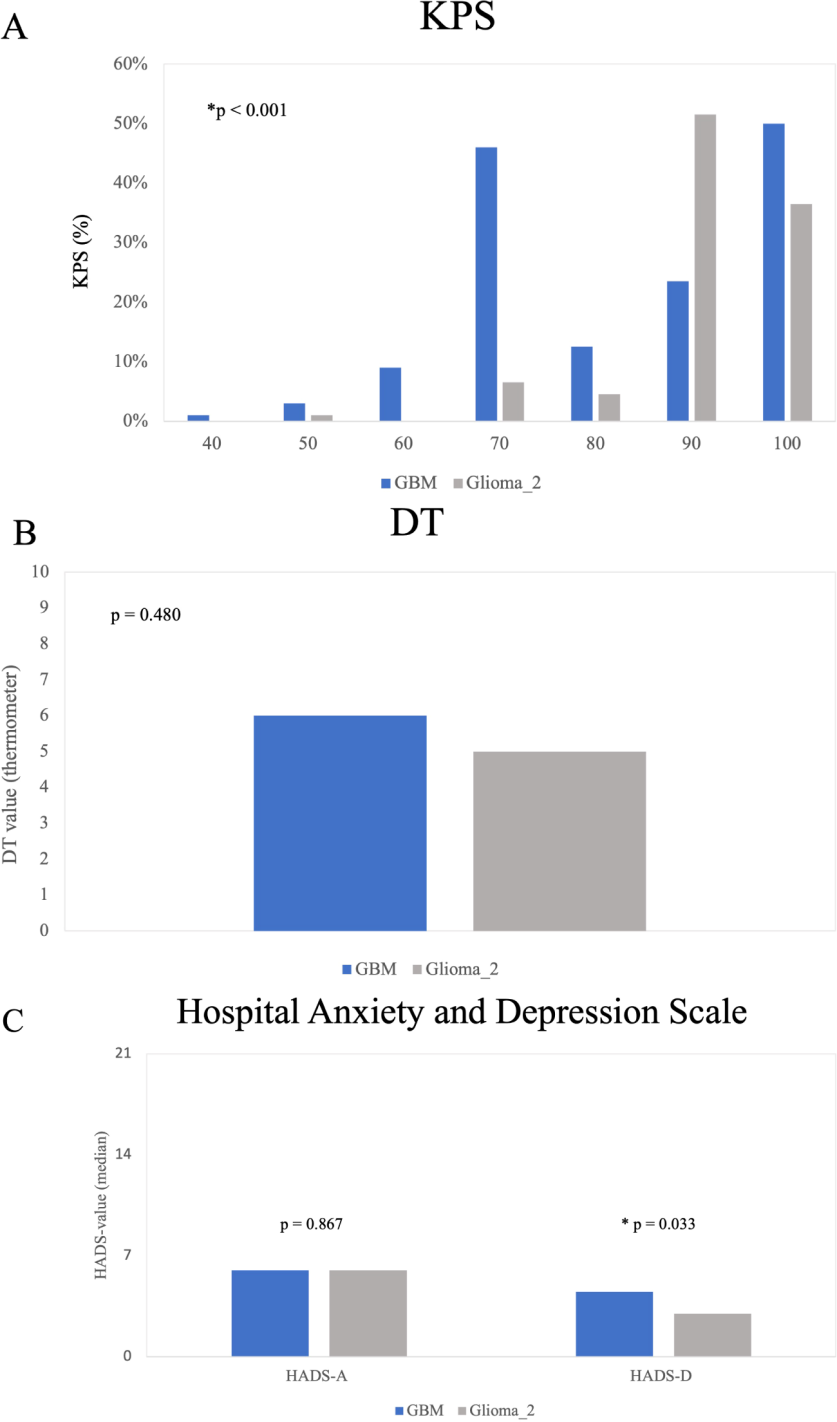
**(A–C)** Results from KPS **(A)**, DT **(B)** and HADS **(C)**, * indicating significant p-values in group comparison, GBM data visualised in blue, Glioma_2 data visualised in grey. **(A)**: Distribution of patients across the KPS scores from 40 to 100 (%), comparing GBM patients with Glioma_2 patients. The median KPS for GBM cohort was 70, while the median KPS for Glioma_2 patients was 90, p < 0.001. **(B)** Median DT results for GBM patients compared to Glioma_2 patients. While the trend indicates higher values for GBM patients, the results remained not statistically significant (p=0.480). **(C)** Median values for HADS-A (left) and HADS-D (right) in GBM patients compared to Glioma_2 patients. There was no significant difference in the median HADS-A scores between the diagnostic groups (p=0.867), while GBM patients report a higher median in HADS-D than Glioma_2 patients (p=0.033).

The KPS was significantly lower in GBM patients compared to Glioma_2 patients, with median KPS scores of 70 for GBM (Mean = 75.7, SD = 12.175) and 90 for Glioma_2 (Mean = 91.13, SD = 9.599); p= < 0.001 (**Figure 1A**). Age significantly predicted KPS in the overall cohort (p < 0.001), but within each diagnosis group, age had no significant impact (GBM: p = 0.175; Glioma_2: p = 0.05). Additionally, KPS was not significantly affected by gender, survey timing, or pre-existing psychiatric conditions.

### DT

Out of 213 patients, 141 (66%) reported a DT score above the cut-off value of 5. The median DT score did not differ significantly between GBM patients (median = 6; mean = 5.43; SD = 2.77) and Glioma_2 patients (median = 5; mean = 5.15; SD = 2.568); p = 0.480 (**Figure 1B**). Among GBM patients, 99 out of 152 (65.6%) had a DT score ≥5, compared to 41 out of 61 (67.2%) Glioma_2 patients, demonstrating high distress levels across both groups. Further analysis showed that gender, family situation, timing of the survey, or pre-existing psychiatric conditions did not have a significant impact on distress levels as measured by the DT.

### HADS

#### HADS-Anxiety

No significant difference in HADS-A scores was observed between GBM and Glioma_2 patients. GBM patients had a median HADS-A score of 6 (mean = 6.72, SD = 4.993), while Glioma_2 patients had a median score of 6 (mean = 6.51, SD = 4.006); p = 0.867 (**Figure 1C**). 38.6% of GBM patients (44 out of 114 patients) reported a HADS-A score of ≥8. Of these, 14.9% (17 patients) had a HADS-A score between 8-10, and 23.7% (27 out of 114 patients) had a HADS-A score of ≥11. In the Glioma_2 group, 38.8% (19 out of 49 patients) reported a HADS-A score of ≥8. Of these, 26.5% (13 patients) had a HADS-A score between 8-10, and 12.2% (6 out of 49 patients) had a score of ≥11.

#### HADS-Depression

A significant difference was found in HADS-D scores between GBM and Glioma_2 patients. The median HADS-D score of the GBM group was 4.5 (mean = 5.43, SD = 4.323), whereas Glioma_2 patients had a median score of 3 (Mean = 4.18, SD = 4.410), p = 0.033 (**Figure 1C**). A HADS-D score of ≥8 was reported in 29.8% (34 out of 114) of GBM patients, compared to 16.3% (8 out of 49) in the Glioma_2 group. In the GBM group specifically, 19.3% (22 patients) had HADS-D scores between 8-10, 10.5% (12 patients) had scores of ≥11 and 4.1% (2 patients) among Glioma_2 patients had scores between 8-10, and 12.2% (6 patients) had scores of ≥11.

### Influencing factors on HADS

#### Timing of survey

**Figure 2:**
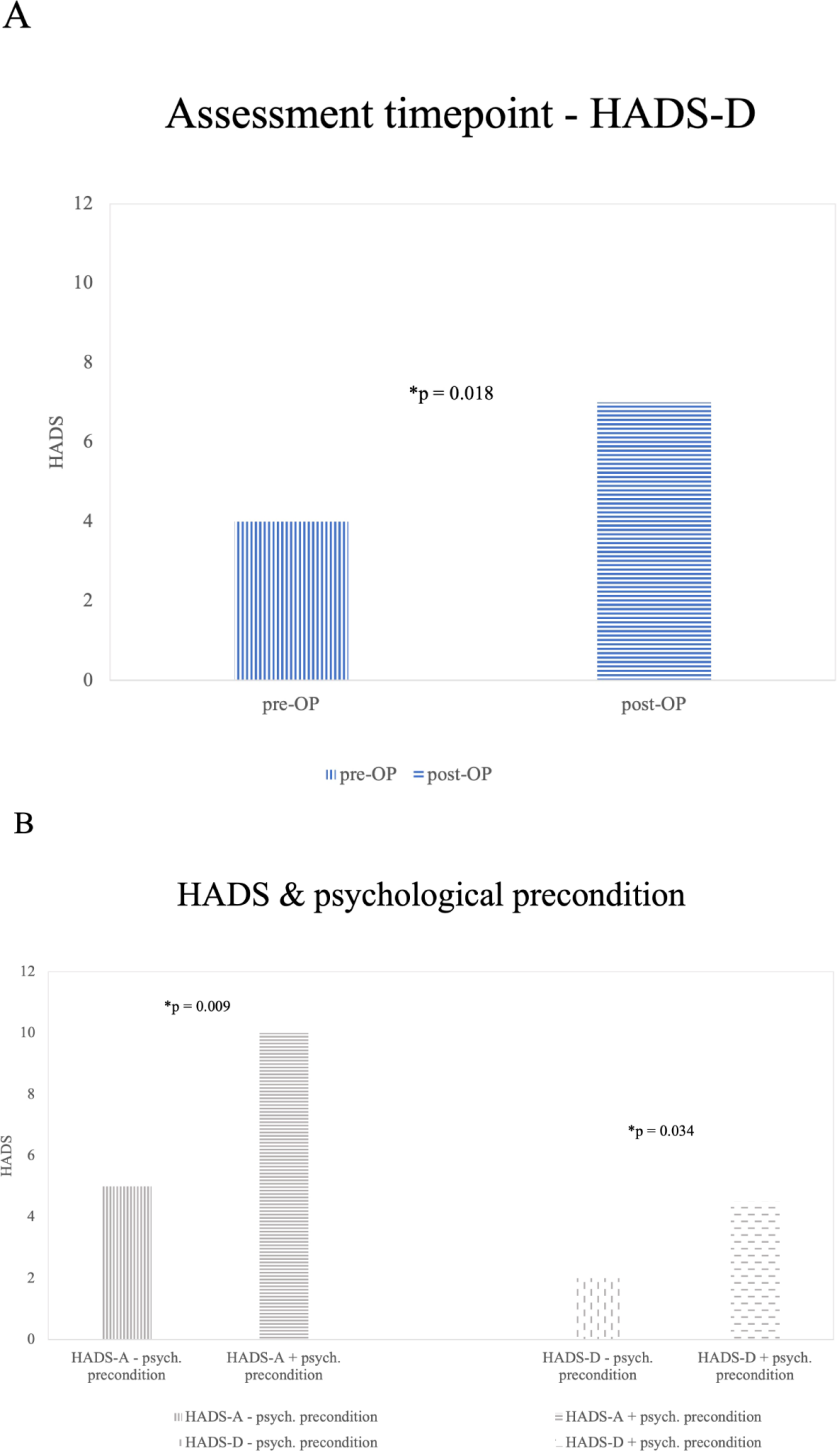
Significant (*) results from influence factors on HADS [**(A)** assessment timepoint, **(B)** psychological precondition]. **(A)** HADS-D and Survey Time Points: This graph shows the median HADS-D scores of GBM patients at different survey time points. GBM patients surveyed pre-operatively reported lower levels of depression compared to those surveyed post-operatively (p=0.018). There were no significant findings in the Glioma_2 group **(B)** Illustrates findings in the Glioma_2 patients group concerning HADS-A and HADS-D differences in patients reporting a history of psychiatric preconditions and patients without a specific history of psychiatric conditions. A significantly (*) higher number of Glioma_2 patients with a history of anxiety (p=0.009) and depression (p=0.034) are affected compared to those without such a history.

GBM patients surveyed preoperatively reported significantly lower depression scores compared to those surveyed postoperatively. The median HADS-D score was 4 preoperatively (mean = 4.68, SD = 3.638) versus 7 postoperatively (mean = 7.21, SD = 5.262). This difference was statistically significant (p = 0.018). Although the median HADS-D scores for both preoperative and postoperative GBM patients were below the cut-off value of 8, a smaller proportion of preoperative patients reported elevated HADS-D scores (23.8%; 19 out of 80) compared to postoperative patients (44.1%; 15 out of 34). The timing of the survey did not reveal significant differences in HADS-A scores (**Figure 2A**).

#### Psychological preconditions and medication

Out of the entire cohort, 34 patients (15.2%) reported a history of psychiatric conditions or ongoing psychotropic medication. HADS data were missing for 8 of these patients. Significant results could only be obtained in the Glioma_2 patients’ group. No significant influence of psychiatric preconditions on HADS-A or HADS-D scores was observed in GBM patients (n=14).

Within Glioma_2 patients, HADS-D scores were significantly higher among those with a history of psychiatric conditions (median = 4.5; mean = 6.33; SD = 4.887) compared to those without (median = 2; mean = 3.49; SD = 4.073; p = 0.034. 33.3% (4 out of 12) reported HADS-D scores above the cut-off of 8, with all these patients indicating HADS-D scores ≥11. In contrast, among Glioma_2 patients without psychiatric conditions, only 10.8% (4 out of 37) reported HADS-D scores ≥8 (5.4% scored between 8-10, and 5.4% reported scores ≥11).

Regarding HADS-A scores among Glioma_2 patients, a history of psychiatric conditions (Median = 10; Mean = 9.33; SD = 4.376) significantly elevated scores compared to those without known psychiatric conditions (median = 5; mean = 5.59; SD = 3.468; p = 0.009. 66.7% (8 out of 12) reported HADS-A scores above the cut-off of 8. In contrast, among Glioma_2 patients without psychiatric conditions, 29.7% (11 out of 37) reported HADS-A scores ≥8. Of these, 24.3% (9 out of 37) scored between 8-10, and 5.4% (2 out of 37) reported scores ≥11. Results are illustrated in **Figure 2B**.

### EORTC-QLQ-C30-BN20 domains of QoL

**Figure 3:**
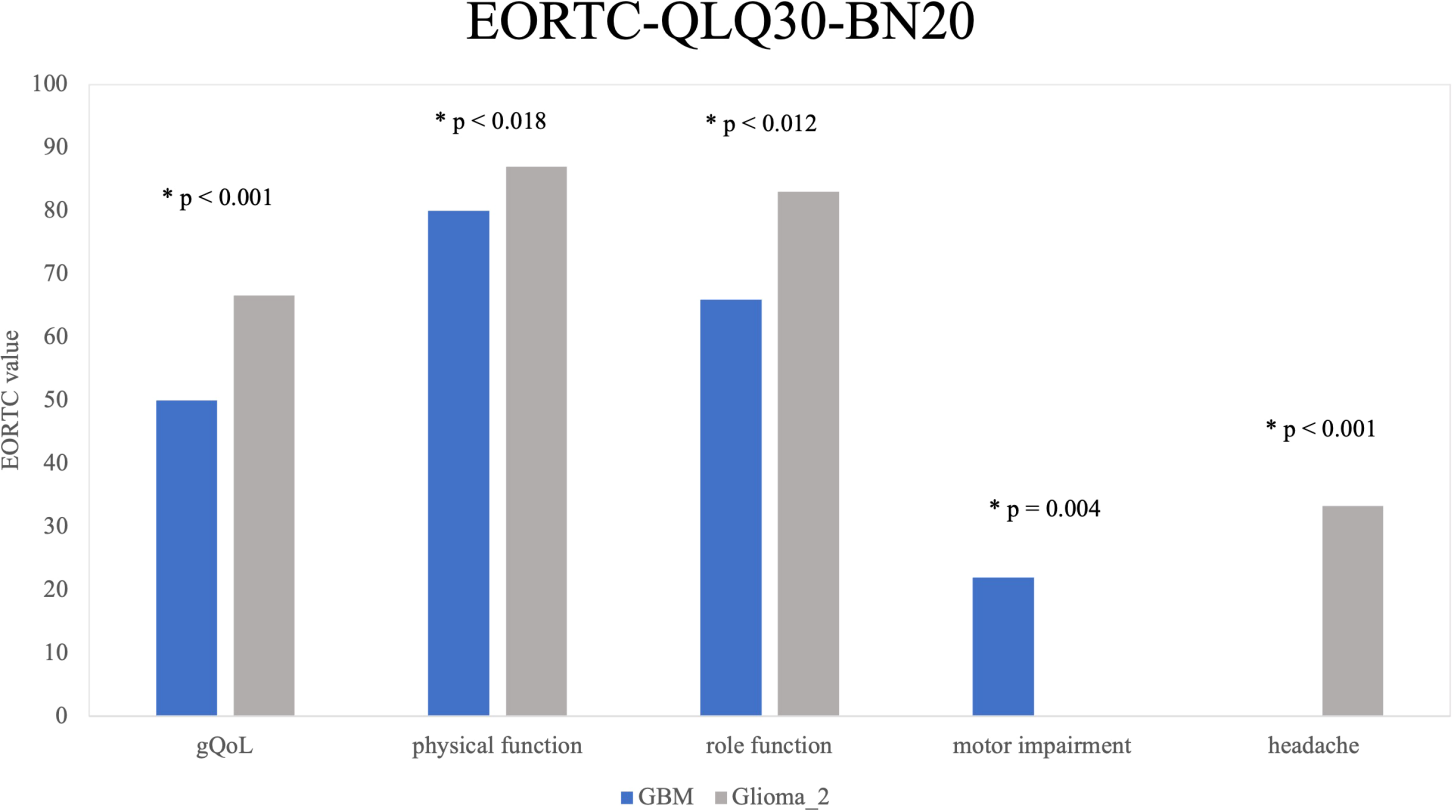
EORTC-QLQ-C30-BN20 by Diagnosis Groups: Five key quality-of-life areas with significant differences between diagnosis groups are shown. Glioma_2 patients showed significantly higher and thus better scores in gQoL (p < 0.001), physical functioning (p = 0.018), and role functioning (p = 0.012) compared to GBM patients. GBM patients report more motor limitations (p = 0.004). Headaches are the only negative aspect more common in Glioma_2 patients than in GBM patients (p < 0.001).

Significant differences were observed within the diagnostic groups. GBM patients reported significantly lower median values in the following domains of QoL: gQoL (GBM median = 50; mean = 50.57; SD = 27.496 vs. Glioma_2 median = 66.67; mean = 64.54; SD = 23.935; p < 0.001), physical function (GBM median = 80; mean = 69.68; SD = 31.055 vs. Glioma_2 median = 87; mean = 81.41; SD = 21.928: p = 0.018), role Function (median = 66; mean = 57.89; SD = 37.093 vs. Median = 83; mean = 72.25; SD = 31.496; p = 0.012). Furthermore, motor function impairments were significantly higher among GBM patients (median = 22; mean = 27.78; SD = 30.275) compared to Glioma_2 patients (median = 0; mean = 15.07; SD = 22.072; p = 0.004). Significant differences were also observed in the subscales of headache (GBM < Gliom_2, p < 0.001) and incontinence (GBM > Gliom_2, p = 0.023). In all other aspects of quality of life assessed by the EORTC-QLQ-C30-BN20, there were no statistically significant differences observed between the diagnostic groups (emotional, cognitive, and social functioning, fatigue, nausea/vomiting, pain, dyspnoea, insomnia, appetite loss, constipation, diarrhoea, financial difficulties, fear of future, visual problems, communication limitations, seizures, dizziness, hair loss, itching). **Figure 3** illustrates significant findings from the EORTC-QLQ-C30-BN20 subdomains.

### Influencing factors of EORTC-QLQ-C30-BN20 domains of QoL

Only the analyses yielding significant results within the GBM or Glioma_2 group are reported.

In our analysis, gender was identified as a significant factor influencing communication limitations in the GBM patients’ group (women > men, median = 33; mean = 35.80; SD = 31.426 vs. median = 16.56; mean = 23.92; SD = 26.982; p = 0.019).

Additionally, there was a significant difference observed in physical function related to the timing of assessment (preoperative median = 86; mean = 73.97; SD = 29.378 vs. postoperative median = 73; mean = 60.44; SD = 32.819; p = 0.01) in GBM patients’ group. Furthermore, visual problems, as a component of physical function, showed significant differences over time (preoperative median = 0; mean = 12.69; SD = 18.783 and postoperative median = 11; mean = 20.43; SD = 24.374; p = 0.045). Finally, pre-existing psychiatric conditions were found to significantly influence aspects of the EORTC-QLQ-C30-BN20.

In the aspect of gQoL, GBM patients with psychiatric distress exhibited significantly lower scores (median = 25; mean = 35.8; SD = 33.029) compared to those without such distress median (50; mean = 52.33; SD = 26.343; p = 0.037).

Similarly, in Glioma_2 patients, gQoL scores were significantly lower at a significance level of exactly 5% among those with psychiatric distress (median = 50; mean = 53.12; SD = 26.68) compared to those without (median = 66.67; mean = 68.51; SD = 21.822; p = 0.05).

## Discussion

The question of QoL is crucial in the treatment of patients diagnosed with glioma. While the implementation of screening has been thoroughly examined in recent years, insights into the timing of necessary interventions or significant differences arising from the diagnoses of LGG compared to a GBM have not been adequately studied. Therefore, we, in this study, specifically investigated whether Glioma_2 patients, among other factors, experience better QoL and lower psycho-oncological distress due to their better prognosis and younger age at the onset of their illness compared to GBM patients. We analysed factors such as gender, psychological predisposition, marital status, and physical constitution to understand their influence.

Key conclusions that can be drawn from our data are that by using unspecific screening tools for non-specific stress in this study, the DT, no significant difference between patients with GBM and those with Glioms_2 could be found. However, concerning the domain of depression, when assessed with the HADS-D, significantly more GBM patients reported depression, whereas anxiety levels assessed using the HADS-A were similar in both groups. To evaluate the patients’ physical functions, the medically assessed KPS and some of the subjectively answered aspects of the EORTC-QLQ-C30-BN20 questionnaire that were used showed that GBM patients already have significantly more physical impairments compared to Glioma_2 patients at the time of diagnosis. Among the other aspects of the EORTC-QLQ-C30-BN20 questionnaire, the overall QoL is notably worse for glioblastoma patients. The complexity of QoL could be illustrated through our study.

Integrating our results with existing published data is challenging due to the limited number of glioma studies using the EORTC-QLQ-C30-BN20 questionnaire. Few studies have referenced this questionnaire. Budrukkar’s work is highly comparable, as it uses baseline data from LGG and HGG patients before adjuvant therapy. However, this study involves an Indian patient cohort with a young average age of under 40 years, presenting a demographic difference from the study presented here. Unlike the cohort analysed here, Budrukkar’s patients with disabilities were assisted in answering the questions, suggesting a more varied physical and cognitive condition among the Indian patients (19). Additional sources of LGG data include the study by Gustafsson (27) and, for HGG data, the study by Osoba (28). The data collection periods in both studies align broadly with this current work. It remains unclear whether, unlike in this study, Gustafsson included patients with recurrences.

Furthermore, a comparison with EORTC-QLQ-C30 values from the general population will be conducted. The data from the United Nations Department of Economic and Social Affairs, providing age-adjusted (18-70 years) surveys of the German average population (N=1006), are particularly suitable for this purpose (29). Given the high variability in EORTC-QLQ-C30 results within the average population, the official reference values from the EORTC-QLQ-C30-BN20 manual and the survey of the German average population from Schwarz’s study are also incorporated (30, 31).

### Distress and emotional function in glioma patients

Glioma patients in this study report elevated levels of non-specific distress, consistent with findings in previous literature (32, 33). Using the DT to compare distress levels between different neuropathological entities, specifically GBM and lower-grade gliomas (Glioma_2), no significant difference is found in our data. This aligns with the literature suggesting the DT’s limited sensitivity to tumour stage across various cancer types (34–36). Contrary to the overall results, a smaller study with a limited LGG cohort (n=8) indicates higher DT scores in HGG patients compared to LGG patients despite generally low DT values reported (37). This discrepancy may reflect the DT’s variable sensitivity depending on the sample size and composition. From the crucial finding of increased stress present regardless of the grading of the diagnosis, it can be concluded that every patient newly diagnosed with a glioma, irrespective of its grading, should be offered psycho-oncological support.

In the EORTC-QLQ-C30-BN20 questionnaire, glioma patients in our cohort reported greater limitations in emotional functioning compared to age-matched controls (19, 29). Consistent with DT findings, no significant differences in emotional function are detected between the diagnosis groups within this study. However, Budrukkar’s study reports better emotional function in LGG patients compared to HGG patients, possibly due to cultural influences. In the study, they observed emotional function is best among older normal population controls, suggesting age-related influences on emotional well-being, as reported by Nolte et al. (29).

GBM patients, who are typically about 25 years older than Glioma_2 patients, might initially have better emotional function relative to their younger counterparts. However, the aggressive nature of GBM potentially reduces their emotional function over time. Another significant aspect of high distress in Glioma_2 patients might be their life situation. Being generally younger, these patients are often engaged in family planning, childcare, and pursuing unfulfilled life goals, contributing to their elevated distress levels.

### Anxiety

In this study, 39% of glioma patients show elevated anxiety levels, with 20% even having scores of ≥11. Compared to other studies, the prevalence of anxiety in our cohort is relatively low (10, 25, 33, 38, 39). A study in 1999 found that anxiety levels in pre-operative brain tumour patients were 20% higher than those reported in this study, indicating potential progress in managing emotional side effects over time (40). Unlike the study, our data did not reveal significant statistical differences in HADS-A scores between different diagnostic groups. Similarly to our data, the study by Bunevicius et al. found no pre-operative differences in HADS-A scores between HGG and LGG at initial diagnosis (38). However, a study by Arnold et al. reports higher anxiety in LGG patients compared to HGG patients, citing the inclusion of many complex cases and a generally high prevalence of depression as contributing factors (41).

### Depression

Unlike anxiety, depression is significantly more reported by GBM patients in this cohort than by Glioma_2 patients. This is evident in both the median HADS-D scores (4.5 vs. 3) and the percentage of patients above the cut-off (29.8% vs. 16.3%). A similar prevalence was found in a study of pre-operative patients at initial diagnosis using the same cut-off values (37% HGG; 10% LGG) (38). However, another study using objective depression screening tests did not find differences based on tumour histopathology (42). Additional studies confirm increased depression in GBM patients compared to LGG patients and other brain tumour patients (38, 40). Conversely, the study by Arnold et al., study shows higher depression scores in LGG patients than in HGG patients, but direct comparison is limited due to different measurement tools. The authors also attribute the high depression prevalence to the inclusion of many complex cases in his study (41). The elevated depression rates among GBM patients may be linked to their shorter survival times, more severe symptoms, and the burdensome adjuvant therapy they undergo.

Comparing the overall HADS-A and HADS-D results for this cohort, anxiety is more prevalent than depression, with a quarter of the patients scoring above the cut-off of ≥8. Bunevicius also observed higher perioperative anxiety compared to depression (38). Following the conclusion of a meta-analysis on glioma and depression, the prevalence in this study falls within the lower reported range (13-53%) for the risk of glioma patients developing depression (43).

### Physical functioning

In this cohort, GBM patients reported significantly worse physical functioning compared to Glioma_2 patients. Similar findings are presented in Budrukkar’s cited study involving HGG and LGG patients. Regardless of the diagnostic group, there is a pronounced reduction in physical function compared to the age-matched average German population (29).

The interpretation of physical limitations in GBM and Glioma_2 patients should consider the average age difference of approximately 25 years between the groups. Age-related declines in physical function are evident in the age-adapted EORTC-QLQ-C30 results for the general population. Glioma_2 patients show fewer differences in physical functioning relative to the younger average population compared to the difference observed between GBM patients and the older average population. This suggests that neuropathology influences physical function, although a statistically valid calculation to confirm this is not feasible due to the lack of literature data. The presence of more severe physical symptoms is expected, given the more rapid and aggressive tumour growth in GBM.

Supporting these results, the EORTC-QLQ-C30-BN20 questionnaire in this study indicates significantly higher scores for motor deficits among GBM patients compared to Glioma_2 patients. Budrukkar’s study also reports pronounced motor deficits, with greater impairment in HGG patients compared to the LGG cohort.

### Clinical assessment using the KPS

When measuring physical condition using the KPS, which is critical for immediate therapy planning, a significant difference between GBM and Glioma_2 patients is confirmed in this study. Even when only pre- and post-operative Glioma_2 patients are included in the calculation, they exhibit significantly better physical condition than GBM patients at initial diagnosis. While the results for GBM patients in this study align with the literature, Glioma_2 patients show better outcomes than previously reported in the literature (44, 45). This discrepancy may be due to early screening and the exclusion of patients undergoing adjuvant therapy or with recurrences. Regardless of the specific cause, older glioma patients and GBM patients are particularly affected by reduced KPS. In this cohort, the median KPS for Glioma_2 patients was 90, indicating normal activity with minimal or mild symptoms. In contrast, the median KPS for GBM patients was 70, suggesting that while patients can still manage self-care, normal activity or participation in the workforce is no longer possible.

### Overall QoL in glioma patients

Our study results indicate a markedly lower subjective QoL for GBM patients compared to Glioma_2. This distribution of gQoL between GBM and Glioma_2 patients aligns with Budrukkar’s findings. Comparing global QoL outcomes across different age groups in the general population reveals a slight decline in global QoL with increasing age. While Glioma_2 patients in this study exhibit similar global QoL levels to their age-matched general population, GBM patients show substantially lower scores than the 60–69-year-old group in the general population (29). Therefore, the diminished gQoL observed in GBM patients cannot be fully attributed to the older average age of these patients. Hickmann reports better gQoL scores for HGG patients compared to the GBM patients in this study and finds no significant difference between HGG and LGG patients, possibly due to varying disease stages within the patient cohort (11). Additionally, Osoba et al. note higher, near-normal gQoL scores for perioperatively surveyed HGG patients (28). The reason for the differences in gQoL outcomes among various HGG cohorts remains unclear.

### Limitations

The challenging measurability of QoL stems from its inconsistent definition and the variety of measurement methods used. This complicates the comparison of data, exacerbated by differently defined cut-off values, various analytical methods and differing survey timings in the literature. The rarity of the disease often results in small and heterogeneous study cohorts. Furthermore, deviations in neuropathological diagnosis based on current criteria cannot be ruled out when compared with older data.

To address these limitations, this study presents results from multiple established measurement methods with precise statistical details, highlighting relevant differences found in the literature. Comprehensive data collection is not fully achieved due to the adaptation of sociodemographic questions during the data collection period. Retrospective data collection is not feasible for deceased patients, meaning that an analysis of additional potential confounders can only be conducted with new data over time. The retrospective character of this study is, therefore, one major limitation. This study cannot entirely rule out the impact of “selection bias,” as patients unable to complete the questionnaires independently were excluded. The impact is considered minimal given the early survey timing and the relatively unaffected patients. It is recognised in recent publications that cognitively impaired individuals tend to report a higher QoL than their caregivers (46). Glioma patients report lower scores in the “cognitive function” aspect of the EORTC-QLQ-C30 compared to the general population, indicating that bias cannot be excluded. Furthermore, the groups were unevenly distributed, and assessment took place at different time points. These facts have to be clarified to contextualise the data. Prospective data collections are needed to reduce possible bias.

## Conclusion

In this study, we analysed the complex field of QoL by analysing data from GBM and Glioma_2 patients. The initial assumption of lower QoL and higher psycho-oncological burden among GBM patients, compared to Glioma_2 patients, was substantiated in key aspects. We conclude from our data that besides a general need for psychooncological screening, especially targeted anxiety management interventions for glioma patients and early screening for depression, especially among GBM patients, should become more standard practice. The EORTC-QLQ-C30-BN20 questionnaire emerged as a comprehensive screening tool, revealing significant differences not only in physical domains but also in other aspects between GBM and Glioma_2 patients. Particularly, gQoL vividly portrayed the poorer state of GBM patients compared to Glioma_2 patients. A substantial influencing factor was a history of psychological burden, reflected in diminished global QoL and increased cognitive impairments among psychologically burdened patients.

In summary, our results advocate for early QoL screening of all glioma patients. The understanding of individual life situations offers targeted support for personal limitations. Due to the known interconnectedness between QoL and survival, QoL should be further implemented as a therapeutic goal, and the results of the present study aim to contribute to this advancement.

## Data Availability

The raw data supporting the conclusions of this article will be made available by the authors, without undue reservation.
