# Supplementary material for: Influence of neuropathological diagnosis on psychooncological distress in neurooncological patients - a retrospective cross-sectional analysis
Source: Front Oncol. 2024 Nov 22;14:1457017. doi: 10.3389/fonc.2024.1457017 (PMC11621086; doi:10.3389/fonc.2024.1457017)
Supplement: Supplementary file 1 [file DataSheet1.pdf]

Supplementary material

Influence of Neuropathological Diagnosis on Psychooncological Distress in Neurooncological Patients - A Retrospective Cross-Sectional Analysis

Franziska Staub-Bartelt[1*], Sarah Obermayr[2], Michael Sabel[1], Marion Rapp[1]

[1]Department of Neurosurgery, University Hospital Duesseldorf, Germany

[2] Kufstein Bezirkskrankenhaus, Endach, Österreich

Corresponding author:

*Franziska Staub-Bartelt

Department of Neurosurgery, Medical Faculty, Heinrich-Heine University Düsseldorf, Germany

Moorenstraße 5

40225 Düsseldorf, Germany

Phone: +49 – 211 8105340

franziska.staub-bartelt@med.uni-duesseldorf.de

Study design: retrospective, single-centre

Keywords: quality of life, glioma, neurooncology, mental health, distress

QUESTIONNAIRES USED IN THE STUDY

- EORTC QLQ-C30 (European Organization for Research and Treatment of Cancer Quality of Life Questionnaire-Core 30): A standardized questionnaire for evaluating the QoL in cancer patients (https://www.eortc.org/app/uploads/sites/2/2018/08/Specimen-QLQ-C30-English.pdf)

The EORTC initially was released in 1986/87 as QLQ-C36, the current version (EORTC QLQ-C30 Version 3.0) includes 30 evaluable questions covering 15 aspects of quality of life. Each aspect is scored on a scale from 0 to 100% [1].

The QoL aspects are categorized into four groups:

1. Five Functional Aspects: Assessed through multiple questions - physical, role, cognitive, emotional, and social functioning.
2. Three Symptom Aspects: Assessed through multiple questions - fatigue, pain, and nausea/vomiting.
3. Six Additional Symptoms: Assessed through single questions - dyspnoea, insomnia, appetite loss, constipation, diarrhoea, and financial difficulties.
4. Global Health and Quality of Life: Two questions assess overall health and quality of life, which can be combined into a global Quality of Life (QoL) score.

Questions on global health and overall QoL are rated from 1 (very poor) to 7 (excellent), while all other questions are rated from 1 (not at all) to 4 (very much). Completing the questionnaire typically takes 11 minutes, with minimal need for assistance. Studies since 1993 have confirmed its reliability and validity [2]. Scores are calculated using a raw score multiplied by a specified weight, resulting in a scale from 0-100 for each of the 15 QoL aspects. High scores reflect high functioning in functional aspects, while lower scores indicate better outcomes for symptom aspects.

- EORTC QLQ-BN20 (European Organization for Research and Treatment of Cancer Quality of Life Questionnaire-Core 30, https://www.eortc.org/app/uploads/sites/2/2018/08/Specimen-BN20-English.pdf).

  In addition to the general cancer questionnaire, there are disease-specific modules. The QLQ-BN20 was designed for brain tumour patients, featuring 20 specific questions. It assesses aspects such as future uncertainty and six typical symptoms. Like the QLQ-C30, responses range from 1 to 4, with scores calculated between 0-100 for each aspect [3]. The EORTC QLQ-C30 and QLQ-BN20 questionnaires are suitable for tracking changes over time. A change of 10 points is considered significant, while a change of more than 20 points is deemed substantial [4].

- HADS (Hospital Anxiety and Depression Scale): An instrument for assessing anxiety and depression in hospital patients: The HADS was developed in 1983 for use in general somatic illnesses. The S3 Guideline recommends HADS for screening psychological distress alongside the Distress Thermometer (DT) [5]. HADS is a concise questionnaire that patients can complete by indicating their agreement or disagreement with statements about their feelings. It consists of 14 questions without somatic symptoms: 7 related to anxiety and 7 to depression. Each question is rated on a scale from 0 (not at all) to 3 (very much). The questionnaire can be completed in 5-10 minutes, and scoring is straightforward. The results provide separate scores for anxiety and depression, which can also be combined to give a general distress score, though this combined score is not used in this study due to the use of DT for general distress. Scores are interpreted in three ranges: 0-7 (normal), 8-10 (borderline), and 11+ (abnormal). A cut-off score of 8 increases sensitivity but reduces specificity, capturing more at-risk patients. Conversely, a cut-off score of 11 reduces false positives and enhances specificity at the cost of sensitivity. The scale has confirmed psychometric properties (objectivity, reliability, and validity [6]). While HADS cannot diagnose depression solely based on self-reported symptoms, elevated scores suggest the need for further evaluation by a specialist.

Example of HADS-assessment questionnaire (from https://www.svri.org/sites/default/files/attachments/2016-01-13/HADS.pdf)

Hospital Anxiety and Depression Scale (HADS)

Tick the box beside the reply that is closest to how you have been feeling in the past week.
Don't take too long over you replies: your immediate is best.

D	A		D	A	
		**I feel tense or 'wound up':**			**I feel as if I am slowed down:**
	3	Most of the time	3		Nearly all the time
	2	A lot of the time	2		Very often
	1	From time to time, occasionally	1		Sometimes
	0	Not at all	0		Not at all
		**I still enjoy the things I used to enjoy:**			**I get a sort of frightened feeling like 'butterflies' in the stomach:**
0		Definitely as much		0	Not at all
1		Not quite so much		1	Occasionally
2		Only a little		2	Quite Often
3		Hardly at all		3	Very Often
		**I get a sort of frightened feeling as if something awful is about to happen:**			**I have lost interest in my appearance:**
	3	Very definitely and quite badly	3		Definitely
	2	Yes, but not too badly	2		I don't take as much care as I should
	1	A little, but it doesn't worry me	1		I may not take quite as much care
	0	Not at all	0		I take just as much care as ever
		**I can laugh and see the funny side of things:**			**I feel restless as I have to be on the move:**
0		As much as I always could		3	Very much indeed
1		Not quite so much now		2	Quite a lot
2		Definitely not so much now		1	Not very much
3		Not at all		0	Not at all
		**Worrying thoughts go through my mind:**			**I look forward with enjoyment to things:**
	3	A great deal of the time	0		As much as I ever did
	2	A lot of the time	1		Rather less than I used to
	1	From time to time, but not too often	2		Definitely less than I used to
	0	Only occasionally	3		Hardly at all
		**I feel cheerful:**			**I get sudden feelings of panic:**
3		Not at all		3	Very often indeed
2		Not often		2	Quite often
1		Sometimes		1	Not very often
0		Most of the time		0	Not at all
		**I can sit at ease and feel relaxed:**			**I can enjoy a good book or radio or TV program:**
	0	Definitely	0		Often
	1	Usually	1		Sometimes
	2	Not Often	2		Not often
	3	Not at all	3		Very seldom

Please check you have answered all the questions

Scoring:
Total score: Depression (D) ___________ Anxiety (A) ______________
0-7 = Normal
8-10 = Borderline abnormal (borderline case)
11-21 = Abnormal (case)

- Distress Thermometer (DT)

  The DT is a multidisciplinary screening tool developed by the National Comprehensive Cancer Network (NCCN) in the USA [7]. The term "distress" was chosen as a non-discriminatory, secular, and broadly understandable term. Distress is defined as "a broad spectrum of unpleasant emotional experiences of a psychological, social, or spiritual nature, ranging from normal feelings of vulnerability, sadness, and fear, to disabling problems such as depression, anxiety disorders, panic, social isolation, and spiritual crisis." The DT is a self-assessment tool using a simple pencil-and-paper questionnaire, designed for ease and "ultra-rapid" completion. Patients indicate their distress level over the past week, including the current day, on an analogue scale depicted as a thermometer, ranging from 0 (no distress) to 10 (extreme distress). Scores ≥ 5 are considered elevated. Additionally, a 36-item problem list can be used to specify the sources of distress (practical, emotional, psychological, family-related), though this option is not used in the study. The DT's psychometric properties have been evaluated in studies, with its objectivity being internationally recognized while its reliability has not been formally tested. According to the manual, its validity has been confirmed through multiple correlations with the HADS [6]. The DT's sensitivity and specificity depend on the chosen cut-off value and the validation instrument used. For instance, sensitivity is higher with a lower cut-off value, and specificity is higher with a higher cut-off. In a German oncological study, sensitivity ranged from 71.2% to 96.8%, and specificity from 40.7% to 62.4%, depending on the threshold. Optimal cut-off values range between 3 and 5 in various studies [8, 9]. This study uses the recommended cut-off value of ≥5 for neuro-oncological patients.

Introduction

FIRST: On the thermometer on the right mark the number (0-10) which best describes how stressed you have felt during the past week, including today.

Extremely stressed

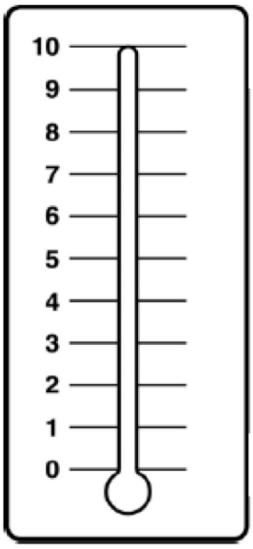

No stress

SECOND: Indicate whether you have had problems in one of the following areas in the past week, including today. Mark YES or NO for each item.

YES	NO		YES	NO	
		**Practical Problems**			**Physical Problems**
❍	❍	Home situation	❍	❍	Pain
❍	❍	Insurance	❍	❍	Nausea
❍	❍	Work/School	❍	❍	Tiredness
❍	❍	Transport	❍	❍	Sleep
❍	❍	Childcare	❍	❍	Getting around/mobility
			❍	❍	Bathing, dressing
		**Family Problems**	❍	❍	Appearance
❍	❍	Relating to your partner	❍	❍	Breathing
❍	❍	Relating to your children	❍	❍	Oral inflammation
			❍	❍	Eating/foods
		**Emotional Problems**	❍	❍	Indigestion
❍	❍	Worry	❍	❍	Constipation
❍	❍	Anxiety	❍	❍	Diarrhea
❍	❍	Sadness	❍	❍	Changes in urination patterns
❍	❍	Depression	❍	❍	Fever
❍	❍	Tenseness	❍	❍	Dry/itchy skin
❍	❍	Loss of interest in everyday activities	❍	❍	Dry/congested nose
			❍	❍	Tingling in hands/feet
			❍	❍	Swelling/bloated feelings
		**Spiritual/religious Concerns**	❍	❍	Memory/concentration
❍	❍	Relating to God	❍	❍	Sexual problems
❍	❍	Loss of faith			

Other problems: ____

NCCN 1.2005 Distress Management Guideline. © National Comprehensive Cancer Network. Alle Rechte vorbehalten. Deutsche Version: Mehnert, A., Müller, D., Lehmann, C., Koch, U. (2006) Die deutsche Version des NCCN Distress-Thermometers - Empirische Prüfung eines Screening-Instruments zur Erfassung psychosozialer Belastung bei Krebspatienten. Zeitschrift für Psychiatrie, Psychologie und Psychotherapie, 54 (3), 213-223.

- KPS: The KPS was introduced in 1949 as an objective tool to evaluate the outcomes of oncological treatments. KPS assesses the physical functioning of patients, particularly their ability to work and care for themselves. Patients are assigned a score ranging from 0 to 100 based on their performance status, as evaluated by medical professionals. For many years, QoL for glioma patients was primarily measured using the KPS [10]. The use of KPS allows for a standardized assessment of the overall health status of patients and contributes to a comprehensive evaluation of their QoL. The first column presents the KPS score from 0-100. The second column contains the corresponding written explanation of the KPS score. A high KPS score indicates a good physical condition. The cut-off for patient independence is set at 70.

KPS in %	Performance
100	No evidence of disease
90	Normal activity, minor signs of disease
80	Normal activity, signs of disease with effort
70	Self-care, unable to carry out normal activity
60	Assistance needed, able to care for most own needs
50	Considerable assistance required
40	Disabled, special care and assistance required
30	Severely disabled
20	Supportive treatment needed
10	Moribund
0	Death

SUPPLEMENTARY DISCUSSION OF FACTORS INFLUENCING THE QOL IN THIS STUDY

In this analysis, gender and pre-existing psychological conditions emerge as particularly influential factors affecting specific aspects of QoL in glioma patients. In contrast, familial status (such as having children or being single or in a relationship) does not show consistent effects. No impact of relationship status or parenthood on diagnosis groups is evident in the KPS and DT scores, nor is there a significant influence on QoL in GBM patients based on familial situation, as measured by HADS and the EORTC-QLQ-C30-BN20 questionnaire. Due to the small cohort size, no definitive conclusions can be drawn about Gliom_2 patients. Existing literature on brain tumour patients also shows inconsistency regarding the emotional burden experienced by singles compared to those in relationships [11-14]. The impact of familial status is largely determined by the quality of interpersonal relationships and specific circumstances such as children's age, their health, caregiving needs, partner relationship quality, partner's health, and social support. These factors should be included in future studies for more accurate results.

Gender

Comparing genders using various measurement tools, the literature often reports higher emotional distress among women compared to men [8, 12, 15, 16]. Our study confirms such tendencies in the DT and HADS, though without statistical significance. The "emotional function" domain of the EORTC-QLQ-C30-BN20 questionnaire also shows no significant gender differences. An analysis of brain tumour patients reveals a significant gender influence on the "emotional well-being" aspect of the SF-36 scale [17]. Meta-analyses present varying results on the influence of gender on QoL, generally indicating that gender affects emotional well-being. Genetic factors and estrogen levels are cited as potential explanations [18]. However, no impact of gender on physical constitution is found in both this study and the literature. In this study, while communicative deficits tend to be influenced by neuropathological diagnosis, a clear gender impact on communication is evident. Female GBM patients experience significantly more communicative limitations than their male counterparts, though comparable literature values are absent. As the EORTC-QLQ-C30-BN20 is a subjective measure, it does not reflect objective communication impairments. Women generally have a higher perceived need for communication, which might explain their heightened perception of limitations.

Psychological Burden

Examining psychological preconditions as confounding factors in this study reveals significantly poorer gQoL outcomes for patients with a history of psychological issues. This result is observed even with a small affected cohort (n=32), across different diagnosis groups, indicating a high need for support among these patients. The impact of pre-existing psychological conditions on the EORTC-QLQ-C30-BN20 questionnaire in brain tumour patients has not been extensively studied in the current literature.

However, the findings are supported by numerous scientific studies that report increased vulnerability to distress, depression, and anxiety in patients with a history of psychological problems [12, 15, 18-20].

In our cohort, no significant impact of psychological preconditions on DT is evident, but HADS-A and HADS-D scores are significantly higher among Glioma_2 patients with pre-existing conditions. The well-known increased susceptibility to depression following previous depressive episodes, regardless of oncological diagnosis, suggests the need for lifelong vigilance for such symptoms [21].

References

1. Fayers, P. and A. Bottomley, *Quality of life research within the EORTC-the EORTC QLQ-C30. European Organisation for Research and Treatment of Cancer.* Eur J Cancer, 2002. **38 Suppl 4**: p. S125-133.
2. Cocks, K., J.R. Wells, C. Johnson, H. Schmidt, M. Koller, S. Oerlemans, G. Velikova, M. Pinto, K.A. Tomaszewski, N.K. Aaronson, et al., *Content validity of the EORTC quality of life questionnaire QLQ-C30 for use in cancer.* Eur J Cancer, 2023. **178**: p. 128-138.
3. Chow, R., N. Lao, M. Popovic, E. Chow, D. Cella, J. Beaumont, H. Lam, N. Pulenzas, G. Bedard, E. Wong, et al., *Comparison of the EORTC QLQ-BN20 and the FACT-Br quality of life questionnaires for patients with primary brain cancers: a literature review.* Support Care Cancer, 2014. **22**(9): p. 2593-2598.
4. Taphoorn, M.J. and A. Bottomley, *Health-related quality of life and symptom research in glioblastoma multiforme patients.* Expert Rev Pharmacoecon Outcomes Res, 2005. **5**(6): p. 763-774.
5. *Leitlinienprogramm Onkologie (Deutsche Krebsgesellschaft, Deutsche Krebshilfe, AWMF): Psychoonkologie, Langversion 2.01, 2022.* p. https://www.leitlinienprogramm-onkologie.de/fileadmin/user_upload/Downloads/Leitlinien/Psychoonkologie/Version_2/LL_Psychoonkologie_Langversion_2.01_Konsultationsfassung.pdf.
6. Herschbach, P.W., J., *Screeningverfahren in der Psycho.* 2010.
7. Goebel, S. and H.M. Mehdorn, *Measurement of psychological distress in patients with intracranial tumours: the NCCN distress thermometer.* J Neurooncol, 2011. **104**(1): p. 357-364.
8. Mehnert-Theuerkauf, A., D. Müller, C. Lehmann, and U. Koch, *Die deutsche Version des NCCN Distress-Thermometers: Empirische Prüfung eines Screening-Instruments zur Erfassung psychosozialer Belastung bei Krebspatienten.* Zeitschrift für Psychiatrie Psychologie und Psychotherapie, 2006. **54**: p. 213-223.
9. Cutillo, A., E. O'Hea, S. Person, D. Lessard, T. Harralson, and E. Boudreaux, *The Distress Thermometer: Cutoff Points and Clinical Use.* Oncol Nurs Forum, 2017. **44**(3): p. 329-336.

10. Liu, R., M. Page, K. Solheim, S. Fox, and S.M. Chang, *Quality of life in adults with brain tumors: current knowledge and future directions.* Neuro Oncol, 2009. **11**(3): p. 330-339.

11. Hu, Y., F. Deng, L. Zhang, K. Hu, S. Liu, S. Zhong, J. Yang, X. Zeng, and X. Peng, *Depression and Quality of Life in Patients with Gliomas: A Narrative Review.* J Clin Med, 2022. **11**(16).

12. Ley, A., M. Kamp, C. von Sass, D. Hänggi, M. Sabel, and M. Rapp, *Psychooncological distress in low-grade glioma patients-a monocentric study.* Acta Neurochir (Wien), 2022. **164**(3): p. 713-722.

13. Rooney, A.G., S. McNamara, M. Mackinnon, M. Fraser, R. Rampling, A. Carson, and R. Grant, *The frequency, longitudinal course, clinical associations, and causes of emotional distress during primary treatment of cerebral glioma.* Neuro Oncol, 2013. **15**(5): p. 635-643.

14. Kaplan, C.P. and M.E. Miner, *Relationships: importance for patients with cerebral tumours.* Brain Inj, 2000. **14**(3): p. 251-259.

15. Randazzo, D. and K.B. Peters, *Psychosocial distress and its effects on the health-related quality of life of primary brain tumor patients.* CNS Oncol, 2016. **5**(4): p. 241-249.

16. Keir, S.T., R.D. Calhoun-Eagan, J.J. Swartz, O.A. Saleh, and H.S. Friedman, *Screening for distress in patients with brain cancer using the NCCN's rapid screening measure.* Psychooncology, 2008. **17**(6): p. 621-625.

17. Bunevicius, A., S. Tamasauskas, V. Deltuva, A. Tamasauskas, A. Radziunas, and R. Bunevicius, *Predictors of health-related quality of life in neurosurgical brain tumor patients: focus on patient-centered perspective.* Acta Neurochir (Wien), 2014. **156**(2): p. 367-374.

18. Ownsworth, T., A. Hawkes, S. Steginga, D. Walker, and D. Shum, *A biopsychosocial perspective on adjustment and quality of life following brain tumor: a systematic evaluation of the literature.* Disabil Rehabil, 2009. **31**(13): p. 1038-1055.

19. Bunevicius, A., V.P. Deltuva, and A. Tamasauskas, *Association of pre-operative depressive and anxiety symptoms with five-year survival of glioma and meningioma patients: a prospective cohort study.* Oncotarget, 2017. **8**(34): p. 57543-57551.

20. Rapp, M., S. Schipmann, K. Hoffmann, R. Wiewrodt, H.J. Steiger, M. Kamp, W. Stummer, D. Wiewrodt, and M. Sabel, *Impact of distress screening algorithm for psycho-oncological needs in neurosurgical patients.* Oncotarget, 2018. **9**(60): p. 31650-31663.

21. McCarron, R.M., B. Shapiro, J. Rawles, and J. Luo, *Depression.* Ann Intern Med, 2021. **174**(5): p. Itc65-itc80.
